# Mechanical Properties of Recycled Aggregate Concretes Containing Silica Fume and Steel Fibres

**DOI:** 10.3390/ma14227065

**Published:** 2021-11-21

**Authors:** Soheil Jahandari, Masoud Mohammadi, Aida Rahmani, Masoumeh Abolhasani, Hania Miraki, Leili Mohammadifar, Mostafa Kazemi, Mohammad Saberian, Maria Rashidi

**Affiliations:** 1 Centre for Infrastructure Engineering, Western Sydney University, Penrith, NSW 2751, Australia; m.mohammadi@westernsydney.edu.au (M.M.); m.rashidi@westernsydney.edu.au (M.R.); 2 Department of Civil and Environmental Engineering, Alaodoleh Semnani Institute of Higher Education, Garmsar 5815, Iran; masoumeh.abolhasani88@gmail.com; 3 Department of Civil Engineering, Iran University of Science and Technology, Tehran 6846, Iran; hania_miraki@alumni.iust.ac.ir; 4 Department of Architectural Engineering, Kerman Branch, Islamic Azad University, Kerman 1167, Iran; leilimohammadifar@gmail.com; 5 GeMMe Building Materials, Urban and Environmental Engineering, University of Liège, 4000 Liège, Belgium; mostafa.kazemi@uliege.be; 6 School of Engineering, RMIT University, Melbourne, VIC 3000, Australia; mohammad.boroujeni@rmit.edu.au

**Keywords:** compressive behaviour, elastic modulus, recycled aggregates, steel fibres, silica fume

## Abstract

In this study, the impact of steel fibres and Silica Fume (SF) on the mechanical properties of recycled aggregate concretes made of two different types of Recycled Coarse Aggregates (RCA) sourced from both low- and high-strength concretes were evaluated through conducting 60 compressive strength tests. The RCAs were used as replacement levels of 50% and 100% of Natural Coarse Aggregates (NCA). Hook-end steel fibres and SF were also used in the mixtures at the optimised replacement levels of 1% and 8%, respectively. The results showed that the addition of both types of RCA adversely affected the compressive strength of concrete. However, the incorporation of SF led to compressive strength development in both types of concretes. The most significant improvement in terms of comparable concrete strength and peak strain with ordinary concrete at 28 days was observed in the case of using a combination of steel fibres and SF in both recycled aggregate concretes, especially with RCA sourced from high strength concrete. Although using SF slightly increased the elastic modulus of both recycled aggregate concretes, a substantial improvement in strength was observed due to the reinforcement with steel fibre and the coexistence of steel fibre and SF. Moreover, existing models to predict the elastic modulus of both non-fibrous and fibrous concretes are found to underestimate the elastic modulus values. The incorporation of SF changed the compressive stress-strain curves for both types of RCA. The addition of steel fibre and SF remarkably improved the post-peak ductility of recycled aggregates concretes of both types, with the most significant improvement observed in the case of RCA sourced from a low-strength parent concrete. The existing model to estimate the compressive stress-strain curve for steel fibre-reinforced concrete with natural aggregates was found to reasonably predict the compressive stress-strain behaviour for steel fibres-reinforced concrete with recycled aggregate.

## 1. Introduction

Concrete is considered the most widely used construction material in the world [1,2,3,4,5,6,7,8,9,10,11,12,13]. Rapid urbanisation due to population growth results in the redevelopment of housing sectors and infrastructures in many cities around the world [14,15,16,17,18]. These redevelopments generate huge amounts of demolition waste due to the destruction of existing infrastructure, such as buildings and bridges [19,20]. New construction activities also generate concrete and building waste. Therefore, a significant amount of construction and demolition (C&D) waste is generated, and only a small amount is recycled in road bases while the rest goes to landfills [21,22,23,24]. Conversely, in many cities, land areas for C&D wastes disposal are scarce, and the landfill levy to dump these C&D wastes is also increasing every year. Hence, the additional cost is paid off by contractors and asset owners. The use of waste materials, such as C&D wastes, as aggregates in concrete is a sustainable and economical practice in the construction industry [23,25,26].

The properties of concrete made by recycled aggregates have been analysed in many research studies [27,28]. Therefore, there is a good understanding of the mechanical and durability properties of recycled aggregate concrete. As a result, the partial replacement of natural aggregates with recycled aggregates in concrete has been adopted in many projects around the world [29,30]. However, previous studies on the use of the recycled coarse aggregates (RCA) in concrete indicate the lower mechanical and durability characteristics of recycled aggregate concrete in comparison with the natural aggregate concrete, which is due to the weaker properties of RCA in comparison to the natural coarse aggregate (NCA). The weaker performance of RCA is due to the presence of attached mortar and inferior interfacial transition zone (ITZ).

Like concrete-containing natural aggregates, recycled aggregate concrete also exhibits brittle behaviour in tension and flexure [31]. Therefore, various fibres are used to reinforce recycled aggregate concrete to improve its mechanical properties [32,33,34,35]. Among many fibres, steel fibre is one of the most effective materials to enhance the tensile strength of recycled aggregate concrete [36,37,38]. Most studies have investigated the improvement in the mechanical properties of recycled aggregate concrete containing steel fibres by measuring the compressive, tensile and flexural strengths.

To mitigate the weaker performance of RCA and make it more comparable to conventional concrete, multiple approaches have been utilized in previous research, including the addition of supplementary cementitious materials (SCMs) such as fly ash, electric arc furnace slag, ground granulated blast furnace slag, and SF [39]. Such SCMs contribute to strength enhancement through eliminating the inferiority of RCA and make it comparable to natural aggregate concrete. For instance, the latent hydraulic property of ground granulated blast furnace slag as well as its pozzolanic characteristics contributes to the mitigation of the adverse mechanical impacts of RCA [40].

In addition to the desirable impact of the mentioned materials, previous research studies indicate the superb performance of SF in the enhancement of mechanical and durability properties of recycled concrete [41]. The addition of SF improves the mechanical and durability properties of recycled aggregate concrete in two ways. First, SF fills out RCA pores, which later improves the microstructure of the interfacial transition zone; second, hydration products fill the micro-cracks initially present in the RCA during crushing [42]. The incorporation of SF also improves the behaviour of fibre-reinforced concrete [43]. However, there is still a need to better understand the stress-strain behaviour of steel fibre-reinforced recycled aggregate concrete in the construction of structures once SF is used as a supplementary cementitious substance [44,45]. While determining the compressive strength is necessary for calculating the strength of structural components, the stress-strain curve is required for evaluating the toughness resistance to determine the ductility of structures made with sustainable materials [46,47,48].

Carneiro et al. [49] measured the compressive stress-strain behaviour of steel fibre-reinforced concrete containing recycled aggregates replaced by 25% of natural aggregates. The results showed that steel fibres affect the stress-strain behaviour of recycled aggregate concrete and increase its toughness. The behaviour of steel fibre-reinforced recycled aggregate concrete under compression was similar to that of fibre-reinforced natural aggregate concrete. However, to maximise the use of RCA in concrete and increase its sustainability, high amounts of RCA as a replacement for natural coarse aggregate are required.

Meesala [50] studied the effects of various types of fibres, such as woollen fibres, glass fibres, and steel fibres, on the mechanical and durability properties of recycled aggregate concretes. The experimental results showed that the incorporation of fibres could significantly improve the mechanical properties of recycled aggregate concrete. However, steel fibres showed the best performance in enhancing the mechanical properties of concrete. In another study [16], the axial stress-strain behaviour of macro-polypropylene fibres reinforced recycled aggregate concrete was investigated. Test results indicated that the peak stress, peak strain, and ultimate strain of concrete specimens increased with an increase in the fibres dosage, and the addition of fibres had a positive effect on the ductility of recycled aggregate concrete. Additionally, it has been reported that lower aspect ratio of fiber could lead to strength reduction [51]. This is due to the weak bond properties between the cement matrix and the fibres at lower aspect ratios. Furthermore, it has been reported that when the aspect ratio is higher than a specific value, with the addition of steel fibres, the ductility increases rather than the strength of concrete [52].

A better understanding of the compressive stress-strain behaviour and elastic modulus of recycled aggregate concrete containing steel fibre, SF, and their combination needs to be accepted by many designers, contractors, and policymakers as a sustainable alternative to conventional concrete. Therefore, the current study aims to evaluate the impacts of steel fibre and SF and their combination on the compressive stress-strain behaviour and elastic modulus of different recycled aggregate concretes. In published research, it is proven that the replacement levels of up to 30% of NCA by RCA does not significantly jeopardise the mechanical properties of concrete. A recent study reported a 5.0–9.3% reduction in compressive strength when different amounts of RCA were utilized [53]. However, due to the poor mechanical properties of RCA, increasing the replacement levels of NCA by RCA to over 30% can adversely affect the strength properties of concrete once no other additives, such as SF, are added into the mixes [16,54,55]. Therefore, the RCA replacement levels in this study were considered at 50% and 100%, and two different types of RCAs sourced from both low- and high-strength concretes were prepared and tested to investigate the improvement in mechanical properties.

## 2. Experimental Program

### 2.1. Raw Materials

**Figure 1:**
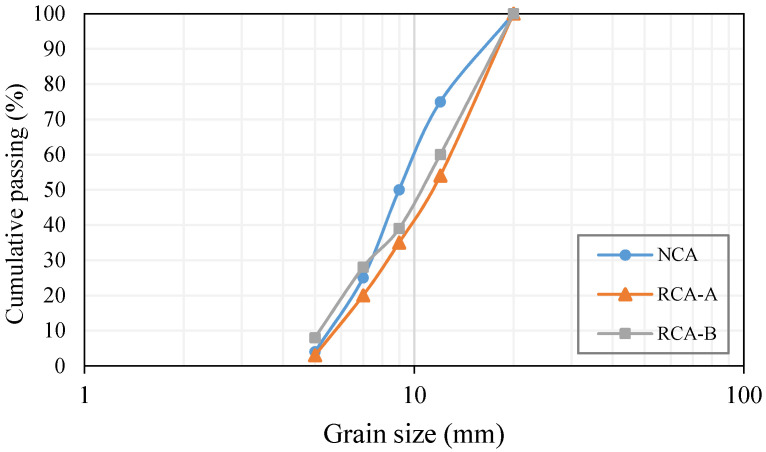
Sieve analysis of natural aggregates and RCAs.

The cementitious materials used in this study were ordinary Portland cement (OPC), equivalent to ASTM Type I, and SF. Their chemical compositions and physical properties are summarised in Table 1. The water quality used to make concrete specimens has a significant impact on concrete strength properties [56,57,58,59]. Therefore, distilled water was utilised for the characterisation tests and tap water for moulding the specimens [60,61,62,63,64,65,66,67,68,69]. Furthermore, the workability of the concrete mixtures was adjusted by using a Sika HRF-2 superplasticiser. Hooked-end steel fibres with a 50 mm length, 0.85 mm diameter, aspect ratio of 60, and tensile strength of 1309 MPa were used. The RCAs, with an angular shape, were obtained by crushing two laboratory concretes with low and high strength levels labelled as “Type A” and “Type B”, with water/cement ratios of 0.60 and 0.40, respectively. The compressive strength of the Type A and B concretes cured for 28 days were 27 MPa and 41 MPa, respectively. The sieve analysis and physical properties of the used aggregates are presented in Figure 1 and Table 2**,** respectively. The attached mortar was obtained according to the thermal method, as recommended by other researchers [70,71]. In this method, before removing all the impurities, such as asphalt, plastics, and bricks, the prepared sample of recycled aggregate (mi) was immersed in water for 2 h to fully saturate the attached mortar. Next, the recycled aggregate sample was placed in a muffle at 500 °C to dry before being immersed in the cold water. This sudden cooling procedure caused cracks and stress generation, leading to easy removal of the mortar from the recycled aggregates. To remove the remaining attached mortar, a rubber hammer was used. Finally, to screen the recycled aggregate sample, a 4 mm sieve was used. Equation (1) was used for calculating the attached mortar:

(1) % attached mortar=(mi − mf)/mi×100

where *mi* and *mf* are the initial and final masses of the sample, respectively.

### 2.2. Mixture Proportions

The mix design of the concrete samples is shown in Table 3. In total, twenty mixtures were prepared, which were divided into four main groups. According to previous research studies [15,71], the optimum percentages of steel fibres and SF that provide sufficient mechanical strength for concrete mixes are 1% (by volume) and 8% (by cement weight), respectively. Therefore, the first group consisted of five control concrete mixtures containing NCA and RCA (including types A and B); the second group included 8% SF used as a partial replacement for OPC. In the third group, 1% steel fibres by volume were added to the mixtures. “S” and “F” were denoted at the beginning of the names of groups two and three, respectively. In the fourth group, the concretes contained 8% SF as a partial replacement for OPC and 1% steel fibres by volume, and “FS” was denoted at the beginning of the mixtures.

A constant water-to-binder ratio equal to 0.4 was used in all the mixtures. Two different RCA contents included the partial replacement of NCA (50% by mass) and full replacement (100% by mass). It should be noted that the replacements were made by mass because the RCA featured a different density compared with the NCA.

The trial-and-error method was used to find the suitable mixing procedure. First, the fine aggregates and binders were mixed using a Hobart mixer for one minute until a homogenous mixture was obtained. Next, half of the mixing water and super-plasticiser were added to the mix of binder and aggregates and were mixed for two minutes. The coarse aggregates and the other half of the water were then added, and the mixing process was resumed for five minutes. Finally, the fibres were added, and the mixing was resumed for five minutes.

### 2.3. Sample Preparation and Test Methods

The freshly mixed concrete was poured into cylindrical moulds with a 100 mm diameter and 200 mm in height to undergo the compressive strength tests and determine the stress-strain curves [45,72]. Next, 24 h after casting, the specimens were demoulded and cured in a basin with 100% relative humidity at 23 °C for 28 days [72,73]. In total, 60 cylindrical samples were prepared, and the compressive strength tests were carried out on them. Three replicate samples were prepared for each test to increase the accuracy of the test results. The uniaxial compressive strength and the stress-strain curves were automatically measured via a data logger connected to a compressive strength test machine with a maximum capacity of 2000 kN, and the loading rate was set to 24 MPa/min.

The slump values of all the mix designs adopted in this study were set to be between 50 mm and 75 mm, which is a reasonable value for practical applications. Moreover, the workability of the samples was slightly reduced while replacing the natural aggregates with both types of recycled concrete aggregates, or by using higher amounts of silica fume. Detailed information regarding the sample preparation, compression tests, and the used standards can be found in recent research studies conducted by the authors [45,74]. The elastic modulus for each specimen was measured by calculating the slope of the linear portion of the compressive stress-strain curve [46]. In other words, the concrete elastic modulus (Es) was calculated from the stress-strain curves according to Equation (2) [75]:

(2) $$E_{s}=\frac{\sigma_{2}-\sigma_{1}}{\varepsilon_{2}-0.005\%}$$

where $\sigma_{2}$ is equivalent to the 40% of the peak load, $\sigma_{1}$ corresponds to the strain at 0.005%, and $\varepsilon_{2}$ is the strain when the stress is equal to $\sigma_{2}$.

## 3. Results and Discussion

### 3.1. Compressive Stress-Strain Behaviour

**Figure 2:**
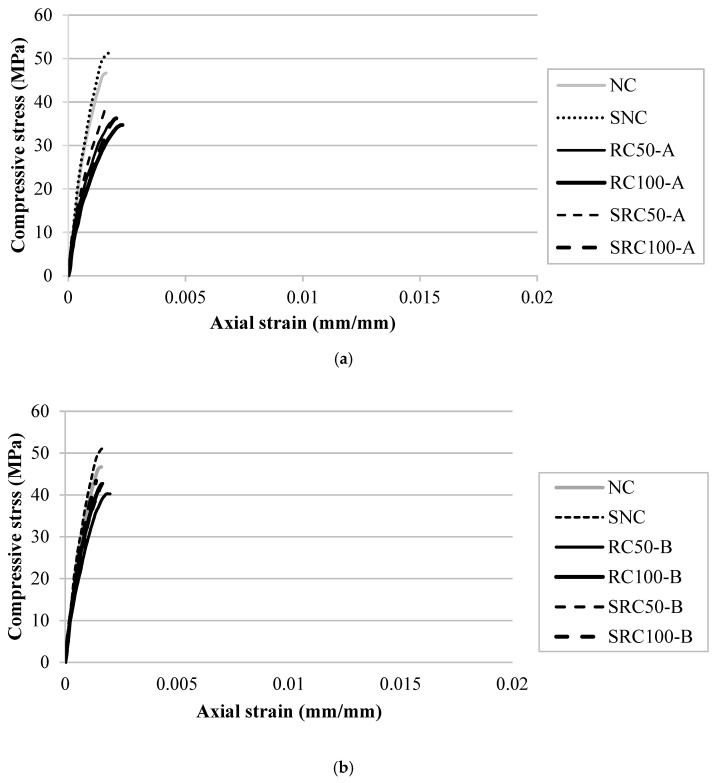
Compressive stress-strain behaviour of non-fibrous concretes containing RCA: (**a**) type A and (**b**) type B.

**Figure 3:**
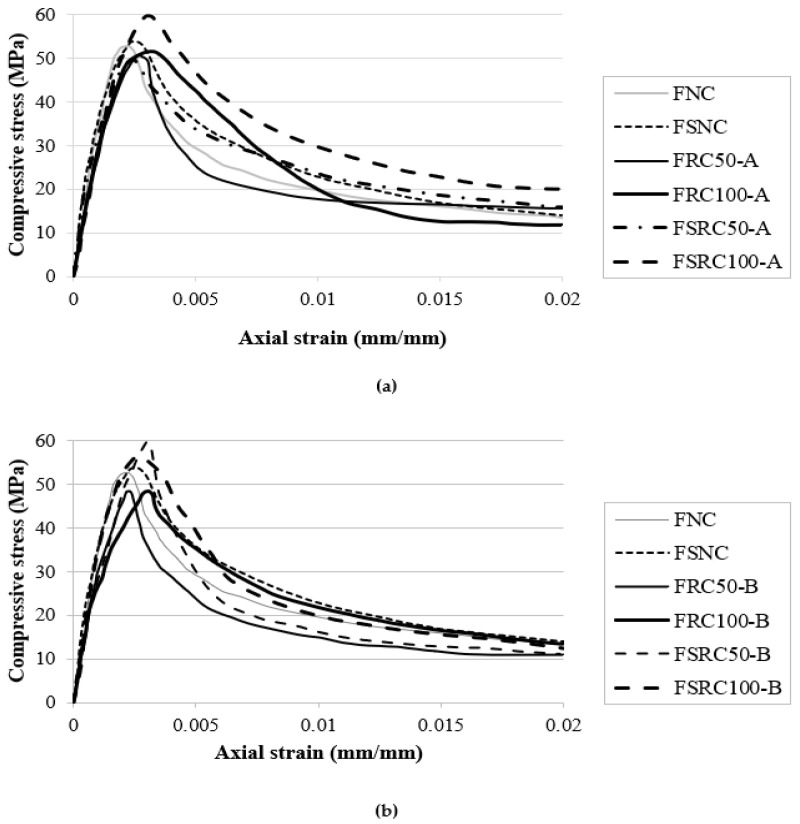
Compressive stress-strain performance of steel fibre-reinforced concretes containing RCA: (**a**) type A, (**b**) type B.

The compressive stress-strain behaviours of the non-fibrous concretes containing RCA types A and B are shown in Figure 2a,b respectively. The stress-strain curves in both types only contain the ascending branch and the peak stress at which the specimen suddenly fractured and failed. This was due to the fact that in the absence of fibre, the samples exhibited brittle behaviour and failed after reaching their peak strength. When comparing the ascending branch of concrete containing NCA with those of the concretes containing RCA types A and B, the slope in the latter cases is less stiff than that of the former. In addition, between concretes containing two types of RCA, the concrete containing RCA type A was less stiff than that of concrete containing RCA type B. The results indicated that the fracture strain of the concretes containing two types of RCA was higher than that of the control concrete containing NCA. This may have been due to the fact that the total ITZ of RCA is higher than that of concrete containing NCA. The increased interfacial zone may give rise to the progressive development of micro-cracks at these interfaces and lead to reduced strength. Naturally, the ITZs of RCA type A, which produced from the low strength concrete, were more extensive than those of the RCA type B, and this may be the reason for the low strength and higher fracture strain of the concrete containing RCA type A. The lower strength of the samples containing RCA type A can also be attributed to the lower strength of the parent concrete. The effect of the SF addition on the ascending branch of the compressive stress-strain behaviour of all the concretes is also shown in Figure 3. Irrespective of coarse aggregate type, the compressive strength and the stiffness of the slopes of all the concretes increased by adding SF. The reason for the improved behaviour in the control concrete containing NCA is pore refinement owing to the particle packing and the formation of additional calcium-silica-hydrate due to the pozzolanic reaction of the SF. The SF also decreased the pores and densified the matrix in the ITZ between the RCAs and the matrix. Previous studies have also reported improvement in the case of ordinary concrete containing NCA [31].

Figure 3 presents the impact of steel fibre inclusion on the compressive stress-strain behaviour of all the concretes. The descending branch of the stress-strain curves was due to the contribution of steel fibres, which increased both the toughness and the ductility of the specimens. The compressive strength and the stiffness of the ascending branch of the stress-strain curve of all the concretes also increased due to the addition of steel fibres. In the case of the concretes containing RCA, the improvement was more prominent. It was observed that the coexistence of steel fibre and SF compensated for the negative effect of RCA in the concretes with the highest strength values of 58.38 and 58.23 MPa for FSRC 100-A and FSRC 50-B, respectively. The results also indicate that the impact of SF was more significant in the fibrous concrete compared with the non-fibrous concretes. This could be tied to the better bonding of steel fibre with the matrix, as observed recently in another study [76]. Additionally, the failure pattern of the fibrous specimens changed from brittle to ductile. The peak strain of the fibrous concretes increased approximately 10 times compared to that of the non-fibrous concretes. The addition of SF also improved the stiffness of the ascending branch slope of the fibrous recycled aggregates concretes, with significant improvement in the case of the concrete containing RCA type B.

**Figure 4:**
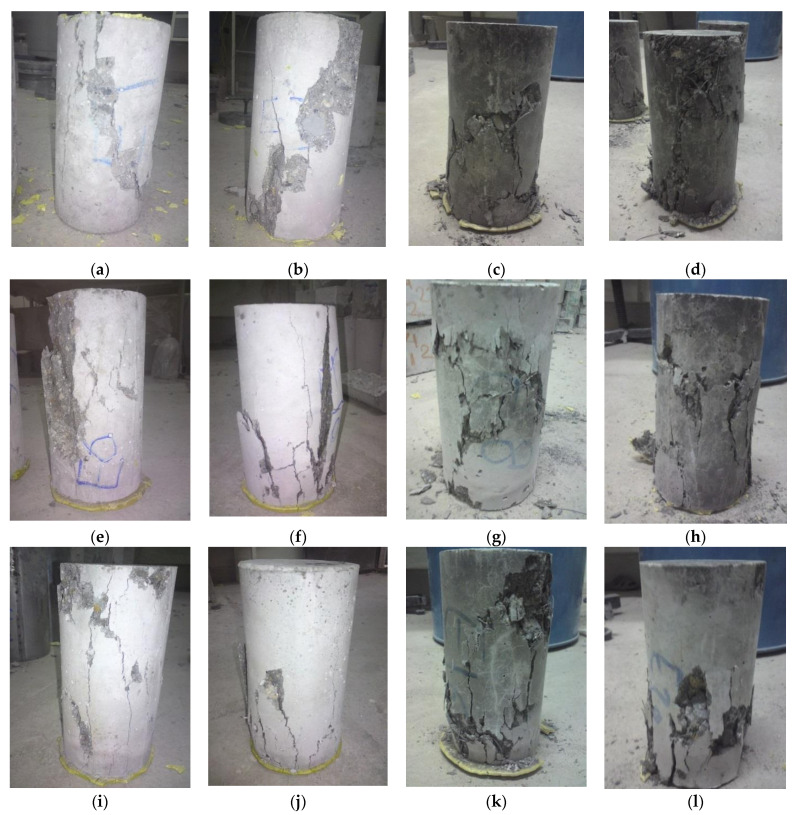
Failure patterns of control concrete and recycled aggregate concretes containing SF, steel fibre, and combination of SF and steel fibre: (**a**) NC; (**b**) SNC; (**c**) FNC; (**d**) FSNC; (**e**) RC100-A; (**f**) SRC100-A; (**g**) FRC100-A; (**h**) FSRC100-A; (**i**) RC100-B; (**j**) SRC100-B; (**k**) FRC100-B; (**l**) FSRC100-B.

The typical failure patterns of all the concrete samples are shown in Figure 4. All the non-fibrous samples exhibited brittle failure, including those containing RCAs. The addition of steel fibres changed the failure pattern of the cylinders from brittle to ductile, as evidenced from the shear-type failure plane in the specimens, which was also similar to the samples prepared with the combination of SF and steel fibres. By comparing the failure patterns of the concretes containing steel fibre with those containing both SF and steel fibres, more minor damage was seen in the latter than in the former.

### 3.2. Modulus of Elasticity

**Figure 5:**
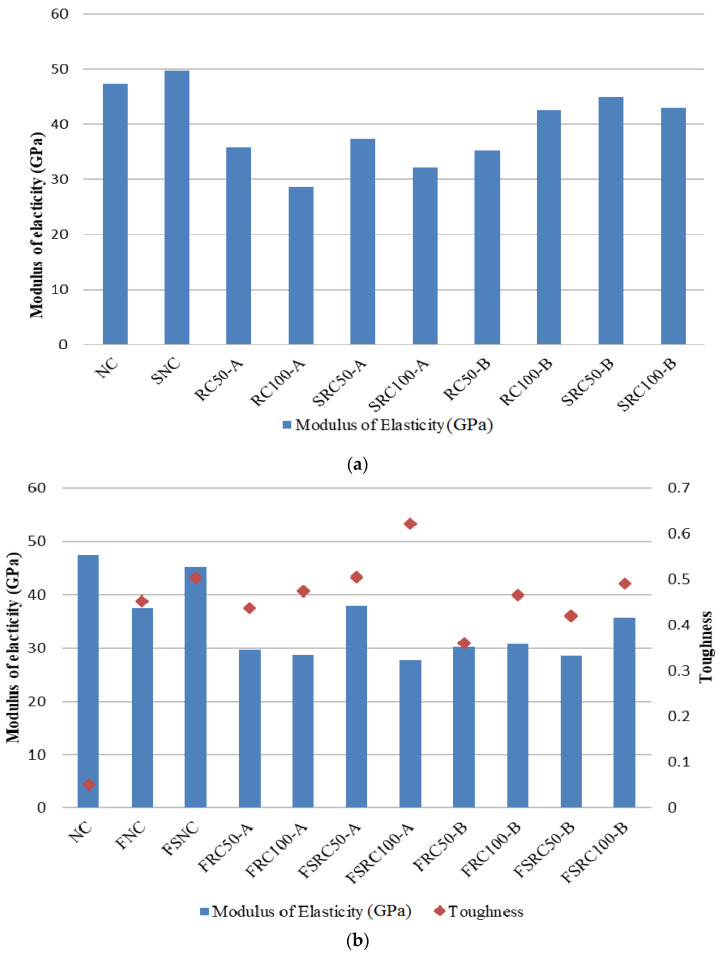
Modulus of elasticity for (**a**) non-fibrous concretes and (**b**) steel fibre-reinforced recycled aggregate concretes.

The calculated elastic modulus values of all the concretes are presented in Figure 5. It can be observed that the modulus of elasticity of the recycled aggregate concretes decreased with an increase in the RCA content. This change could have been due to the lower elastic modulus of the RCA than that of the NCAs and the weaker ITZ of the RCA. Similar results were achieved by Xiao et al. [75] and Salem and Burdette [77]. The replacement with RCA at 50% of both types reduced the modulus of elasticity by about 25%. With 100% replacement of the NCA with RCA types A and B, the elastic modulus decreased by about 40% and 10%, respectively, compared to that of normal concrete. The addition of SF increased the modulus of elasticity of the mixtures in comparison to conventional concrete. This increase could have been due to the pozzolanic activity of SF, which improved the ITZ of the concrete and thus enhanced the modulus of elasticity. Similar results were also reported by Corinaldesi and Moriconi [42]. The addition of steel fibre reduced the elasticity modulus of both the recycled aggregate concretes. For instance, through the introduction of steel fibres, the elasticity modulus of the RC50-A sample was reduced by approximately 19% (from 36.37 to 29.46 GPa in the FRC50-A sample). These results are in line with the findings of Altun et al. [78], who concluded that the modulus of elasticity decreases by increasing the percentage of steel fibre volume. However, the combination of steel fibre and SF had no significant impact on the mixtures containing recycled aggregates type A, but reduced the modulus of elasticity by about 18% in the mixtures containing recycled aggregates type B.

**Figure 6:**
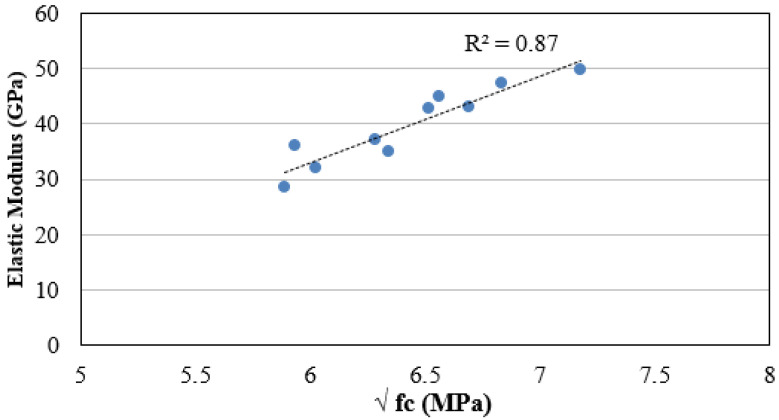
Relationship between elastic modulus and compressive strength of non-fibrous recycled aggregate concretes.

A correlation between the compressive strength and the modulus of elasticity of the non-fibrous recycled aggregates concretes was established, as shown in Figure 6. A reliable correlation was obtained with R^2^ equal to 0.87. The measured elastic modulus values were compared with those predicted by existing models for both the non-fibrous and steel fibre-reinforced concretes to examine the feasibility of using existing models. In the case of the non-fibrous concrete, the models proposed by Warner et al. [79] and Thomas and Ramaswamy [80] for steel fibre-reinforced concrete were considered and compared with the measured values.

**Figure 7:**
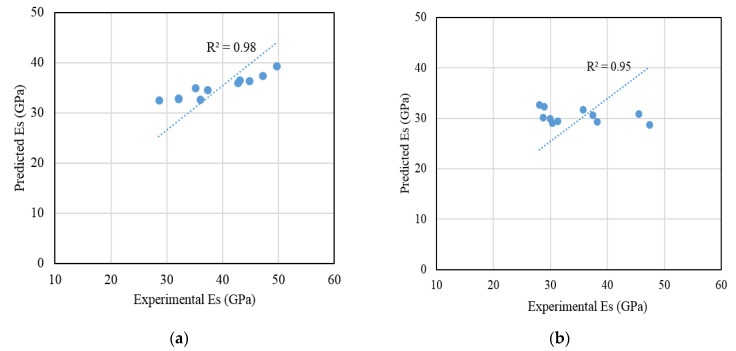
Comparison between experimentally measured elastic modulus and predicted modulus from: (**a**) AS 3600 for non-fibrous recycled aggregate concretes; and (**b**) for steel fibre-reinforced recycled aggregate concretes [80].

Figure 7a,b show the correlations between the experimentally measured and the model-predicted elastic modulus of non-fibrous and steel fibre-reinforced recycled aggregates concretes, respectively. A good correlation can be seen in both cases, with the slight deviation of a few experimentally measured elastic modulus values.

The effect of SF addition on the toughness of both types of steel fibre-reinforced recycled aggregates concretes was calculated from the area under the compressive stress-strain curve in each concrete. The results are summarised in Figure 5b. The toughness values of all the non-fibrous samples were less than 0.1; hence, they are not indicated in the Figure. The toughnesses of the steel fibre-reinforced recycled aggregate concretes containing RCA’s types A and B were comparable with those of the steel fibre-reinforced concrete containing NCA. The addition of SF improved the toughness of both steel fibre-reinforced recycled aggregate concretes such that the toughness increased about 31% (from 0.47 in FRC100-A to 0.62 in FSRC100-A). Similar results were observed in the case of concrete containing NCA. This could have been due to the densification of the ITZ of the steel fibre in the cement matrix, which improved the steel fibre bond in the matrix and hence better post-peak ductility in the concrete.

**Figure 8:**
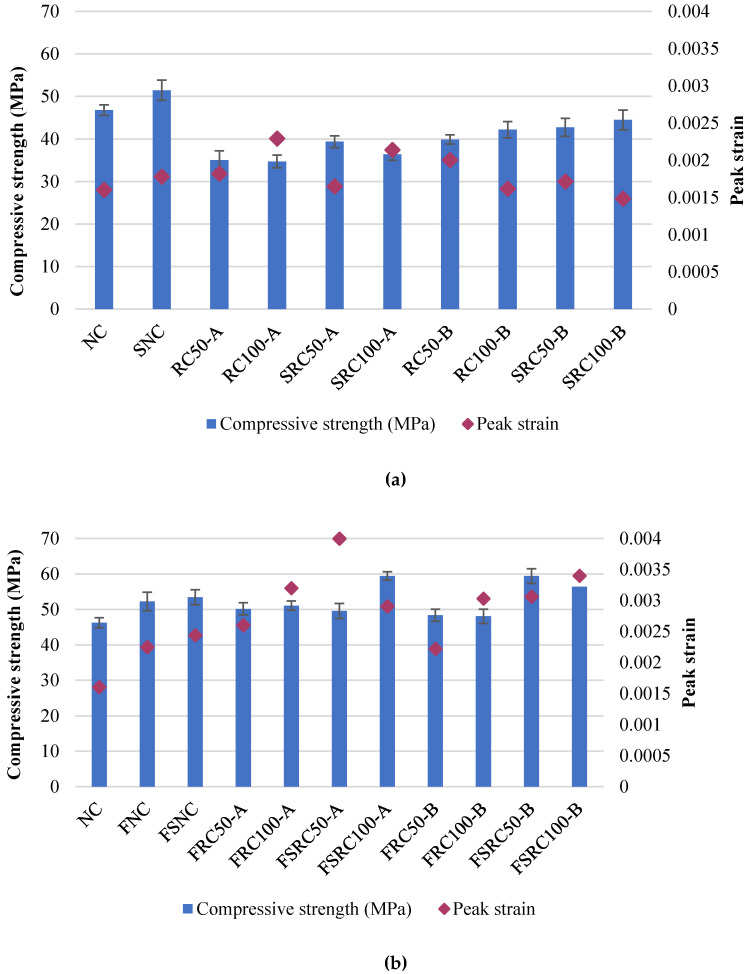
Impact of SF and steel fibre and coexistence of steel fibre and SF on the peak strain and compressive strength of recycled aggregate concretes. (**a**) non-fibrous concretes and (**b**) steel fibre-reinforced recycled aggregate concretes.

The consequences of adding SF on the peak compressive strain and compressive strength of both non-fibrous and fibrous recycled aggregates concretes are shown in Figure 8.

As shown in Figure 8, the peak strain of both types of recycled aggregates concretes was slightly decreased due to the addition of SF. However, in the case of the steel fibres reinforced recycled aggregates concrete, a significant improvement in the peak strain was observed. This improvement can be attributed to the bridging of micro-cracks by the steel fibres. The addition of SF led to a slight improvement in the peak strain of the steel fibre-reinforced recycled aggregate concretes; however, this amount was not very insignificant.

### 3.3. Modelling of Stress-Strain Behaviour of Recycled Aggregates Concretes Containing Steel Fibre and Combination of Steel Fibre and SF

The prediction of compressive stress-strain behaviour of concrete helps to model the structural behaviour of concrete structures. Various models that predict the compressive stress-strain behaviour of concrete containing natural aggregates and fibre-reinforced concretes can be found in previous research, Ezeldin and Balaguru [81] proposed the following model Equation (3) to predict the compressive stress-strain behaviour of ordinary concrete containing steel fibres:

(3) $$\frac{f_{c}}{f_{cf}}=\frac{\beta^{\frac{\varepsilon_{c}}{\varepsilon_{co}}}}{\beta -1+{\left(\frac{\varepsilon_{c}}{\varepsilon_{co}}\right)}^{\beta }}$$

(4) $$\beta =1.093+0.7132\;{\left(RI\right)}^{-0.926}$$

(5) $$RI=V_{f}\frac{l}{ø}$$

where $f_{cf}$ is the compressive strength of fibre concrete; *ε_co_* is the strain corresponding to the compressive strength ($f_{c}$), and $\varepsilon_{c}$ is the strain value in the compressive stress-strain curve. The value β is the material parameter and RI is a reinforcing index combining the effect of the steel fibre volume fractions, where *V_f_* is the volume fraction of fibers, and l and ø are the length and diameter of fibers, respectively [82].

**Figure 9:**
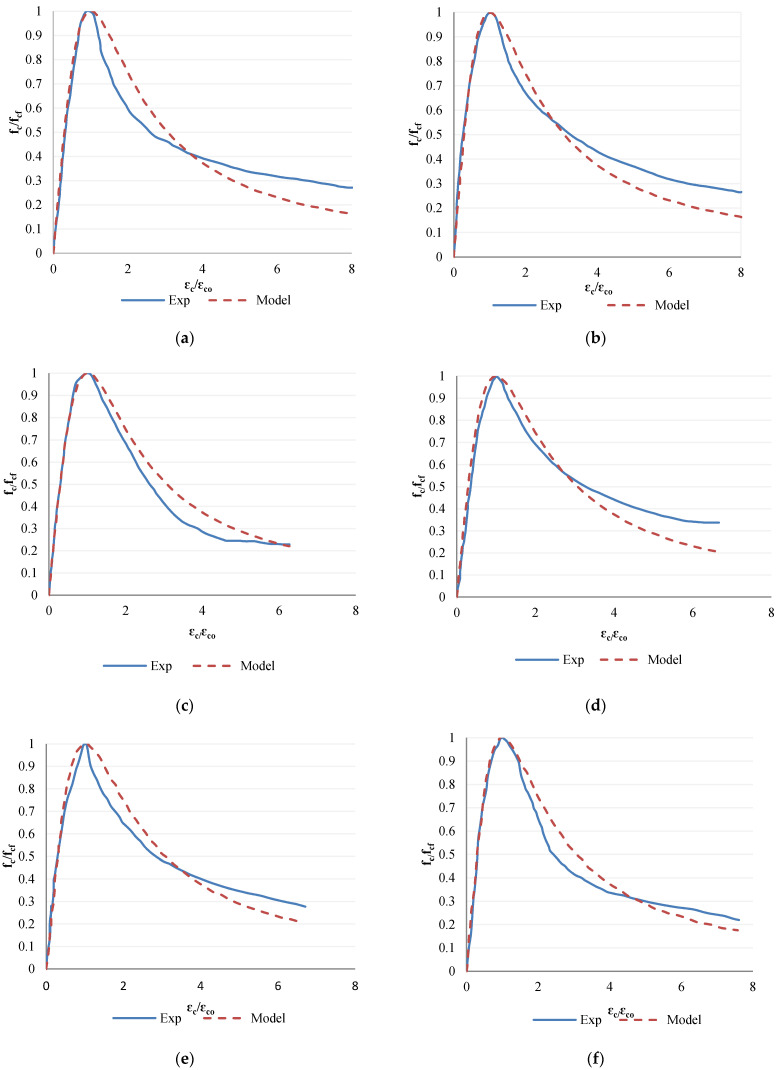
Comparison between the experimental compressive stress-strain curve of steel fibre-reinforced RAC and the predicted model for steel fibre-reinforced NAC proposed by Ezeldin and Balaguru (1992): (**a**) FNC, (**b**) FSNC, (**c**) FRC100-A, (**d**) FSRC100-A, (**e**) FRC100-B, (**f**) FSRC100-B.

The comparison between the experimental compressive stress-strain curve of RCA and that predicted by the aforementioned model proposed by Ezeldin and Balaguru [81] for ordinary concrete containing steel fibres is shown in Figure 9. The model for steel fibre-reinforced concrete containing natural aggregates agrees well with the ascending branch of the stress-strain curve for all recycled aggregates concretes. However, a slight variation in the post-peak behaviour between the model predicted and the experimentally observed curve for recycled aggregates concretes can be seen. Nevertheless, the existing model proposed for steel fibre-reinforced ordinary concrete can be used to predict the compressive stress-strain behaviour of steel fibre-reinforced recycled aggregates concretes even with RCA from different grades of concrete.

## 4. Conclusions

The effects of steel fibres, silica fume (SF), and the combined use of steel fibres and SF on the mechanical properties of recycled aggregate concretes containing 50% and 100% recycled coarse types aggregates (RCA), sourced from both low- and high-strength concretes, were investigated. The following main conclusions were drawn based on the experimental and prediction studies:

1. The discrete addition of SF and steel fibre slightly increased the compressive strength of concretes containing both types of RCA. The combined use of SF and steel fibre significantly improved the compressive strength of recycled aggregates concretes, especially with RCA sourced from high-strength concrete. Similar behaviour was also observed in both recycled aggregate concretes in the case of peak strain.
2. The addition of SF slightly increased the elastic modulus of both recycled aggregate concretes; however, a significant improvement was observed due to the addition of steel fibre and a combination of steel fibre and SF. Existing models underestimate the elastic modulus of both non-fibrous and fibrous concretes at higher magnitudes.
3. The addition of SF improved the ascending branch of the compressive stress-strain curve of the concretes containing both types of RCA. No significant changes in the ascending branch of the compressive stress-strain curve were observed due to the addition of SF in the recycled aggregate concretes containing steel fibre. The addition of steel fibres and the combined addition of SF and steel fibre significantly improved the post-peak ductility of the recycled aggregate concretes of both types, with the most significant improvement, in the case of RCA, sourced from the low-strength parent concrete.
4. The existing model reasonably predicts the compressive stress-strain behaviour of steel fibre-reinforced concrete containing both natural aggregates and recycled aggregates. This indicates the applicability of the existing model for steel fibre-reinforced recycled aggregates concretes with and without SF.

For future research studies, it is recommended to explore the effects of different water/cement ratios on the same mix designs. The investigation of the impact of using other types of fibres on the engineering properties of the mix designs adopted in this research is also suggested.

## Figures and Tables

**Table 1:** Characteristics of cementitious materials [71].

Properties	Cement	Silica Fume
SiO_2_	21.66	90.01
Al_2_O_3_	4.21	1.29
Fe_2_O_3_	3.10	1.09
CaO	63.41	-
MgO	2.82	1.80
SO_3_	2.61	-
Loss of ignition	0.81	-
Relative density, g/cm^3^	3.11	2.20
Specific surface, cm^2^/g	2950	20.700

**Table 2:** Physical characteristics of the aggregates.

Type of Aggregates	Crushing Value (%)	Density (kg/m^3^)	Attached Mortar (%)	Water Absorption (%)
RCA (A)	27.3	2440	25.34	4.45
RCA (B)	27.1	2470	33.51	4.07
SAND	-	2510	-	0.91
NCA	26.5	2630	-	0.47

**Table 3:** Mixture design of concrete samples.

Group	Mix Code	Cement (Kg/m^3^)	Water/Binder	Steel Fibres (Kg/m^3^)	SF (Kg/m^3^)	Sand (Kg/m^3^)	RCA (Kg/m^3^)	NCA (Kg/m^3^)	SP (Kg/m^3^)
Control	NC	380	0.40	-	-	910	-	910	2.30
	RC50-A	380	0.40	-	-	910	455	455	2.30
	RC100-A	380	0.40	-	-	910	910	-	2.30
	RC50-B	380	0.40	-	-	910	455	455	2.30
	RC100-B	380	0.40	-	-	910	910	0	2.30
Silica fume	SNC	350	0.40	-	30	910	-	910	2.30
	SRC50-A	350	0.4.0	-	30	910	455	455	2.30
	SRC100-A	350	0.40	-	30	910	910	-	2.30
	SRC50-B	350	0.40	-	30	910	455	455	2.30
	SRC100-B	350	0.40	-	30	910	910	0	2.30
Steel fibre	FNC	380	0.40	78	-	900	-	900	4.40
	FRC50-A	380	0.40	78	-	900	450	450	4.40
	FRC100-A	380	0.40	78	-	900	900	-	4.40
	FRC50-B	380	0.40	78	-	900	450	450	4.40
	FRC100-B	380	0.40	78	-	900	900	0	4.40
Steel fibre and silica fume	FSNC	350	0.40	78	30	900	-	900	4.40
	FSRC50-A	350	0.40	78	30	900	450	450	4.40
	FSRC100-A	350	0.40	78	30	900	900	-	4.40
	FSRC50-B	350	0.40	78	30	900	450	450	4.40
	FSRC100-B	350	0.40	78	30	900	900	-	4.40

F: concrete containing steel fibre, S: concrete containing silica fume, NC: normal concrete, FS: concrete containing steel fibres and silica fume, RC: recycled aggregate concrete, FSRC100-B: steel fibre-reinforced concrete containing 100% RCA and silica fume.

## Data Availability

Data are available upon reasonable request from the corresponding authors.
